# Clinical and sensitization profile in peach allergy due to LTP sensitization

**DOI:** 10.3389/falgy.2024.1477364

**Published:** 2024-12-03

**Authors:** Zambrano Ibarra Gabriela, Rodríguez Mazariego M. Elena, López Tovar Carlos, Blanco López Marta, Baeza Ochoa M. Luisa

**Affiliations:** Gregorio Marañón Health Research Institute, Allergy Department, Gregorio Marañón General University Hospital, Madrid, Spain

**Keywords:** lipid transfer protein, food allergy, LTP allergy, LTP syndrome, clinical severity

## Abstract

**Background:**

Lipid transfer proteins (LTP) are associated with a wide range of severity of allergic reactions. However, the risk factors associated with this severity are not fully understood.

**Objectives:**

To describe the clinical characteristics of peach-allergic patients due to LTP sensitization and analyze the relationship between the severity of the reactions and patients’ sensitization profiles.

**Methods:**

A retrospective study of peach-allergic patients was performed. Patients were classified into LTP-monoallergic (only peach allergy) or LTP-Syndrome (peach allergy and allergy to other plants-foods related with LTP). Symptoms with Rosaceae family and other related plant foods, skin prick tests (SPTs), and IgE values were recorded.

**Results:**

Seventy-one patients were included, 46.5% suffering from anaphylaxis, 32.3% from urticaria angioedema, and 21.2% from oral allergy syndrome. Six had monoallergy to LTP and 65 LTP syndrome. Clinical severity showed no differences according to peach SPT wheal size or *Pru p 3* IgE levels. We also found no differences between the components of LTP-containing foods analyzed, the number of LTPs recognized, and the severity of symptoms. However, anaphylaxis was more frequent in patients with concomitant allergies to ≥3 groups of plant foods.

**Conclusions:**

LTP syndrome was the most common presentation in patients with Rosaceae food allergy. The severity of the reactions was not related to peach SPT wheal size or sIgE levels to Pru p 3, but concomitant allergies to ≥3 plant food groups could be a good marker of severity.

## Introduction

Lipid transfer protein (LTP) allergy may present as an allergy to a single allergen source or as an LTP syndrome, involving two or more taxonomically unrelated LTP food allergens (1, 2), with heterogeneous degrees of cross-reactivity between different fruits, plant foods, and pollen. Allergic reactions to LTP range from mild oral allergy syndrome to severe anaphylaxis. Nevertheless, some sensitized individuals may remain asymptomatic and not develop LTP allergy (1).

LTP to peach *Pru p 3* is the most frequent primary food allergen causing food-induced anaphylaxis in the Mediterranean area (1). Peach allergy related to nsLTP sensitization exhibit a heterogeneous profile regarding sensitization patterns and symptom severity. This variability may be influenced by coexisting pollen allergies although the role of pollen remains controversial (2–4). However, a high structural homology has been described between Pru p 3, artemisia (Art v 3) and plane tree (Pla a 3) (3, 5).

In LTP allergies, the identification of biomarkers associated with clinical severity is essential for diagnosis and treatment. However, there are some unresolved issues, such as geographical differences, the influence of co-sensitizations to other non-specific. Currently, there is a clear need to identify the patient's LTP profile. In this regard, allergy profiles are analyzed using the available LTPs (foods and pollens). A recent investigation (6) proposes a study plan for patients with LTP allergy with the determination of levels of IgE specific to Pru p 3, Jug r 3, Ara h 9 and Cor a 8. It has been shown sensitization to Pru p 3, Ara h 9 or Cor a 8 was prevalent among people with food allergy and systemic reactions had higher values of Pru p 3, Ara h 9 and Cor a 8 than patients with oral allergy syndrome (OAS) (7).

In our work, these components allow us to better define the sensitization profile, but no LTP component or the number of components recognized showed an association with the severity of symptoms.

On the other hand, it has been identified that the pollen LTP components Art v 3 and Pla a 3 (6, 8–10), showed a clear association with food symptoms, due to their cross-reactivity with predominantly food LTPs. Although we did not specifically test for pollen LTPs, detection of the complete pollen extract of mugwort and plane tree was frequent among in our population and was associated with more severe symptoms. This aligns with previous studies suggesting that pollen sensitization can influence symptoms severity in patients allergic to LTPs (3) although with variable results (6, 8, 11, 12). Among the most relevant pollen types from an allergenic point of view and with the highest concentration in the Community of Madrid are grass pollen, olive, cypress and plane tree (13).

## Methods

This was a retrospective study on adult peach-allergic patients sensitized to LTP that were referred to the Allergy Department of Gregorio Marañón General University Hospital (Madrid, Spain) from January 2020 to July 2021. Patients ≥18 years old with a convincing history of allergic reactions after exposure to peach, and with a positive SPT and/or IgE to *Pru p 3* were included.

Data regarding patient personal history of atopy, symptoms with other plant foods, co-factors, and skin tests were recorded. SPTs with commercial LTP-enriched peach extract (50 μgr of *Pru p 3* enriched, ALK-Abello,Madrid, Spain), profilin, common inhaled allergens, and plant food allergens (LETI lab, Roxall,Bilbao,Spain) were conducted based on the patient's medical history. Prick-by-prick tests and/or open oral challenge tests (OCT) with fresh food were performed to confirm clinical reactivity, except for cases at risk of severe reaction.

In addition, total IgE and sIgE to peach *Pru p3* were measured in all cases. According to the clinical history, sIgE from the complete plant food extract, and sIgE to other LTPs available in our center (i.e., peanut (*Ara h 9*), hazelnut (*Cor a 8*), wheat (*Tri a 14*) and apple (*Mal d 3*) were determined by ImmunoCAP following the manufacturer's recommendations (ImmunoCAP, Thermo-Fisher Scientific Phadia, Uppsala, Sweden). A value >0.35 kUA/L was considered a positive result. Patients with sensitization to storage proteins were not included.

Peach-allergic patients were classified according to the number of LTP allergenic sources into two groups: monoallergy to LTP: individuals who have allergic reactions only with peach and LTP-syndrome: patients who react to peach and at least another plant- food containing LTP.

Quantitative variables are expressed as mean and standard deviation (SD) or median and range, whereas qualitative variables are described as frequencies and percentages. Quantitative and qualitative variables were compared using the Mann–Whitney and Chi-square tests, respectively. Statistical significance was set at a bilateral alpha value of 0.05. All statistical analyses were performed using SPSS software (version 18).

## Results

Seventy-one peach-allergic patients were included, with a mean age of 34.5 (range 17–65) years, 40 (61%) of them were women. All patients were sensitised to *Pru p 3*. Respiratory allergies were detected in 62 patients (87.3%): rhinitis (*n* = 57, 87.6%) and asthma (*n* = 5, 83.3%).

Patients were classified: 6 (8.4%) as monoallergy to LTP and 65 (91.5%) as LTP syndrome. In monoallergic group, 1 (16.6%) had oral allergy syndrome (OAS), 2 (33.3%) Urticaria and 3 (50%) anaphylaxis. From the polyallergic, 30 (46.1%) had anaphylaxis, 21 (32.3%) had urticaria and 14 (21.5%) had OAS.

Anaphylactic reactions were classified following the Food Allergy Severity Score (FASS) with ordinal formats (oFASS) (14). According to the severity of anaphylaxis, LTP syndrome was classified as follows: 15 patients with grade 3 (10 with gastrointestinal symptoms, 5 rhinitis and 3 conjunctivitis), 12 with grade 4 (12 bronchospasms, 4 gastrointestinal symptoms) and 3 with grade 5 (2 neurological and 2 cardiovascular symptoms). While LTP-monoallergy group had 3 patients with grade 4 (3 bronchospasms 1 gastrointestinal symptoms). All patients had urticaria in both groups.

As can be seen in Table 1, their main demographic characteristics, sensitisation profile to inhalants allergens and the severity of symptoms showed no differences between monoallergy to LTP and LTP-syndrome.

**Table 1:** Clinical characteristics of the two groups of patients in the study.

	LTP-syndrome (*n* = 65)	Monoallergy to LTP (*n* = 6)	*p*
Sex (female), n (%)	40 (61)	3 (50)	0.3
Age (years), mean (range)	32 (17–65)	30.5 (17–53)	0.2
Respiratory Allergy, *n* (%)			
Rhinitis	57 (91.5)	5 (83.3)	0.5
Asthma	50 (88.7)	3 (50)	0.1
Pollen sensitization, *n* (%)	57 (87.6)	5 (83.3)	0.5
Grass polen	46 (70.7)	5 (83.3)	0.4
Olive	40 (60.1)	4 (66.6)	0.5
Plane tree	47 (72)	3 (50)	0.2
Mugworth	26 (40)	4 (66.6)	0.2
Peach- symptoms, *n* (%)			
Anaphylaxis	30 (46.1)	3 (50)	0.5
Co-factors-induced anaphylaxis	14 (21.5)		
Urticaria/AE	21 (32.3)	2 (33.3)	0.6
OAS	14 (21.5)	1 (16.6)	0.6
SPT (mm), mean (range)			
Anaphylaxis	6 (4–12)	5 (4–6)	0.4
OAS	5 (3–12)	6 (3–14)	0.1
Urticaria/AE	6 (3–12)	7 (3–14)	0.5
Total IgE (KU/L), mean (range)	573 (140–1,230)	54 (0.46–74.4)	0.4
IgE Pru p3 (KU/L), mean (range)			
Anaphylaxis	5.03 (0.5–35.1)	6.56 (1.02–6.64)	0.5
OAS	5.81 (0.85–74.4)	12.6	0.3
Urticaria/AE	4.95 (0.46–54)	9.97 (2.55–17.4)	0.2
Sensitization to components, *n* (%)			
*Ara h 9*	49 (75.3)	3 (50)	0.1
*Cor a 8*	45 (69)	4 (66.6)	0.4
*Mal d 3*	33 (50.7)	2 (33.3)	0.3
*Tri a 14*	9 (13.8)	1 (16.6)	0.6
Sensitization to profilin, n (%)	17 (26.1)	2 (33.33)	0.2
Anaphylaxis	7 (10.7)	0 (0.0)	ns
Urticaria/AE	5 (7.6)	1 (16.6)	ns
OAS	5 (7.6)	1 (16.6)	ns
Sensitization to PR-10, n (%)	5	0	ns
Anaphylaxis	1 (1.5)	0	ns
OAS	1 (1.5)	0	ns
Urticaria/AE	2 (3)	0	ns
SPT to other plant foods containing LTPs, *n* (%)			
Walnut	35 (53.8)	3 (50)	0.5
Peanut	34 (52)	3 (50)	0.6
Apple	34 (52)	2 (33.3)	0.3
Hazelnut	32 (49)	2 (33.3)	0.3
Almond	12 (18.4)	0	ns
Banana	13 (20)	0	ns

LTP, lipid transfer protein; OAS, oral allergy syndrome; SPT, skin prick test; Urticaria/AE, urticaria and/or angioedema.

Additionally, the SPT wheal area diameter was similar among patients with anaphylaxis, OAS, and urticaria in both groups. Regarding total IgE levels, despite being higher in patients with LTP syndrome, the differences were not statistically significant compared to patients with monoallergy to LTP. Moreover, IgE values to *Pru p 3* did not show statistically significant differences by clinical severity in both groups.

Sensitization to profilin was more frequent in patients with LTP syndrome, especially in those with anaphylaxis, but no statistically significant differences were found Table 1. The frequency of sensitization to PR−10 in our population was low in both groups.

Most patients were sensitized to other plant foods containing LTP, being walnuts, peanuts, apple and hazelnuts the most frequent Table 1.

The molecular components of pollen LTP (i.e., *Art v 3* or *Pla a 3*) were not measured in our study, but in LTP-syndrome we found that sensitization to mugwort (*n* = 26) and plane tree (*n* = 47) were more frequent in patients with anaphylaxis than in patients with milder reactions such as oral allergy syndrome (OAS) and urticaria (*p* = 0.04 and *p* = 0.03 respectively) Table 2.

**Table 2:** Clinical data in LTP-syndrome: sensitisation profile and severity.

	OAS (*n* = 14)	Urticaria (*n* = 21)	Total (*n* = 35)	Anaphylaxis (*n* = 30)	*p*
Sensitization to pollens containing LTP, *n* (%)					
Plane tree	10 (71.4%)	10 (47.6)	20 (57.1)	27 (90)	**0**.**03**
Mugworth	7 (50%)	11 (52.3)	18 (51.4)	8 (26.6)	**0**.**04**
sIgE median kU/L					
sIgE Pru p 3	5.6	7.06		15.9	0.08
sIgE Ara h 9	2.8	2.6		3.75	0.4
sIgE Cor a 8	1.5	2.4		3.3	0.11
sIgE Mal d 3	3.5	4.9		7	0.8
sIgE Tri a 14	0.6	3.8		5.7	ns
Sensitization to components, *n* (%)					
Ara h 9	11 (78.5)	16 (76.1)	27 (77)	22 (73.3)	0.7
Cor a 8	7 (50)	17 (80.9)	24 (68.5)	21 (70)	0.8
Mald 3	7 (50)	8 (38)	15 (42.8)	18 (60)	0.1
Tri a 14	2 (14.2)	4 (19)	6 (17.1)	3 (10)	0.3
Number of sensitized components, *n* (%)					
2	2 (14.2)	4 (19)	6 (17.1)	3 (10)	0.6
3	3 (21.4)	9 (42.8)	12 (34.2)	10 (33.3)	0.2
4	3 (21.4)	4 (19)	7 (20)	10 (33.3)	0.4
5	3 (21.4)	3 (14.2)	6 (17.1)	5 (16.6)	0.4
NP^a^	3	1	4	2	

Bold values are statistically significant.

^a^ Not performed.

*Pru p 3* sIgE levels were higher in anaphylactic group than patients with oral allergy syndrome (OAS) and urticaria, followed by *Cor a 8, Ara h 9* and *Mal d 3* but we did not find a significant correlation Table 2. Sensitization to *Ara h 9, Cor a 8, Mal d 3, Tri a 14* or the number of recognized components were not related to the severity of the reaction.

Although the number of anaphylaxis was lower in participants sensitized to profilin the difference was not statistically significant. However, in patients with LTP syndrome, anaphylaxis was more frequent if they were co-sensitized to ≥3 plant food groups (*p* = 0.04) Supplementary Table S3. It is important to note that given the location of our hospital (central area of Madrid), our population is probably not particularly exposed to peach pollen and there are no fields or cultivation areas nearby, but we do not know if there could be any case in relation to their profession or hobbies.

## Discussion

Few studies have investigated the relationship between peach SPT wheal size, sIgE levels to *Pru p3*, and severity of clinical reactions, but most provide contradictory results (2, 15, 16). For instance, an Italian study found a positive correlation between sIgE *Pru p 3* levels and the severity of reaction (16). Other studies showed no significant differences in *Pru p 3* IgE levels in patients with systemic symptoms (15). Finally, another study concluded that the IgE level of peach LTP is related to increasing number of foods other than Rosaceae (2). However, in our study, we did not find a relationship between the severity of the reaction and the levels of sIgE to *Pru p 3* or peach SPT wheal size.

Some authors have investigated severity biomarkers, like Gador Bogas et al. (8), who described two population groups from different Spanish regions and concluded that co-sensitization to profilin might be the most useful biomarker of a less severe reaction. Nevertheless, in our study, patients with LTP syndrome showing the most severe reactions were also sensitized to profilin. Thus, profilin does not seem to play a relevant role in our patients.

Currently, there is a clear need to identify the patient's LTP profile. In this regard, allergy profiles are analyzed using the available LTPs (foods and pollens). A recent investigation (6) proposes a study plan for patients with LTP allergy with the determination of levels of IgE specific *to Pru p 3, Jug r 3, Ara h 9* and *Cor a 8.* It has been shown sensitization *to Pru p 3, Ara h 9* or *Cor a 8* was prevalent among people with food allergy and systemic reactions had higher values of *Pru p 3, Ara h 9* and *Cor a 8* than patients with oral allergy syndrome (OAS) (7).

In our work, these components allow us to better define the sensitization profile, but no LTP component or the number of components recognized showed an association with the severity of symptoms.

On the other hand, it has been identified that the pollen LTP components *Art v 3* and *Pla a 3* (6, 8, 10), showed a clear association with food symptoms, due to their cross-reactivity with predominantly food LTPs. Although we did not specifically test for pollen LTPs, detection of the complete pollen extract of mugwort and plane tree was frequent among in our population and was associated with more severe symptoms. This aligns with previous studies suggesting that pollen sensitization can influence symptoms severity in patients allergic to LTPs (3) although with variable results (6, 8, 11). Among the most relevant pollen types from an allergenic point of view and with the highest concentration in the Community of Madrid are grass pollen, olive, cypress and plane tree (13).

Recently, an increased incidence of anaphylaxis has been found in individuals sensitized to several plant food groups containing LTP (17, 18). In our study, anaphylaxis was more common in patients with LTP syndrome who were sensitized to ≥3 plant food groups. Therefore, we hypothesize that the number of LTP-containing plant foods could be a risk factor for severe reactions.

Our study has some limitations, such as its retrospective nature, a small sample size, a possible selection bias and a wide range in the number of patients among both groups (monoallergy to LTP and LTP syndrome).

Hence, more studies are required in larger cohorts and different geographic areas to expand our knowledge of diverse populations with different environmental exposures. Nevertheless, this study could provide a better understanding of LTP involvement in the central area of Spain.

In summary, in our study, LTP syndrome was the most common presentation in patients with peach allergy sensitized to LTP. The severity of the reactions was not related to the levels of sIgE to *Pru p 3*, or cosensitisation to profilin. However, we could consider the number of plant foods containing LTP as a possible marker of severity.

## Data Availability

The raw data supporting the conclusions of this article will be made available by the authors, without undue reservation.

## Supplementary material

Supplementary Table S3 Severity of the reaction according to the number of LTP-containing plant foods groups and profilin sensitization in LTP-syndrome.
