# Supplementary figures and images for: Clinical and sensitization profile in peach allergy due to LTP sensitization
Source: Front Allergy. 2024 Dec 3;5:1477364. doi: 10.3389/falgy.2024.1477364 (PMC11649636; doi:10.3389/falgy.2024.1477364)

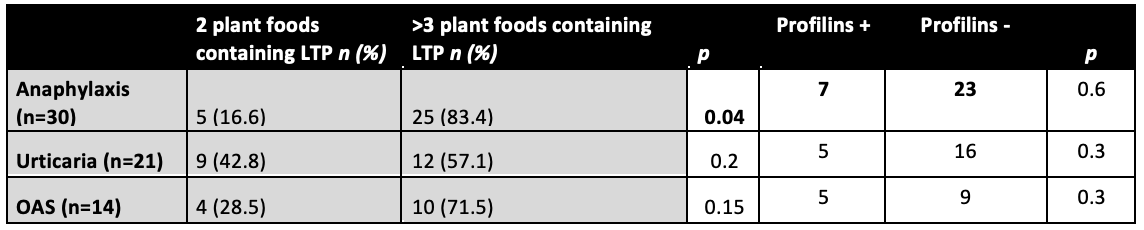

Supplement: Supplementary Table S3 [file Image1.jpeg]
